# Human rhinovirus infection causes different DNA methylation changes in nasal epithelial cells from healthy and asthmatic subjects

**DOI:** 10.1186/1755-8794-7-37

**Published:** 2014-06-19

**Authors:** Peter McErlean, Silvio Favoreto, Fabricio F Costa, Junqing Shen, Jihan Quraishi, Assel Biyasheva, Jocelyn J Cooper, Denise M Scholtens, Elio F Vanin, Maria F de Bonaldo, Hehuang Xie, Marcelo B Soares, Pedro C Avila

**Affiliations:** 1 Department of Medicine, Feinberg School of Medicine, Northwestern University, Chicago, IL, USA; 2 Department of Preventive Medicine, Feinberg School of Medicine, Northwestern University, Chicago, IL, USA; 3 Department of Pediatric, Feinberg School of Medicine, Northwestern University, Chicago, IL, USA; 4 Cancer Biology and Epigenomics Program, Lurie Children’s Memorial Hospital Research Center, Chicago, IL, USA

## Abstract

**Background:**

Mechanisms underlying the development of virus-induced asthma exacerbations remain unclear. To investigate if epigenetic mechanisms could be involved in virus-induced asthma exacerbations, we undertook DNA methylation profiling in asthmatic and healthy nasal epithelial cells (NECs) during Human Rhinovirus (HRV) infection *in vitro*.

**Methods:**

Global and loci-specific methylation profiles were determined via Alu element and Infinium Human Methylation 450 K microarray, respectively. Principal components analysis identified the genomic loci influenced the most by disease-status and infection. Real-time PCR and pyrosequencing were used to confirm gene expression and DNA methylation, respectively.

**Results:**

HRV infection significantly increased global DNA methylation in cells from asthmatic subjects only (43.6% to 44.1%, p = 0.04). Microarray analysis revealed 389 differentially methylated loci either based on disease status, or caused by virus infection. There were disease-associated DNA methylation patterns that were not affected by HRV infection as well as HRV-induced DNA methylation changes that were unique to each group. A common methylation locus stood out in response to HRV infection in both groups, where the small nucleolar RNA, H/ACA box 12 (SNORA12) is located. Further analysis indicated that a relationship existed between SNORA12 DNA methylation and gene expression in response to HRV infection.

**Conclusions:**

We describe for the first time that Human rhinovirus infection causes DNA methylation changes in airway epithelial cells that differ between asthmatic and healthy subjects. These epigenetic differences may possibly explain the mechanism by which respiratory viruses cause asthma exacerbations.

## Background

Although the genetic, phenotypic and transcriptional aspects of asthma are well characterized, epigenetic studies of asthma remain in their infancy. Epigenetic mechanisms including DNA methylation, non coding RNAs (e.g. microRNA) and histone modifications have been linked to the development of asthma [1,2]. However, the role of epigenetic mechanisms in virus or other environmentally induced episodes of asthma exacerbations remains unknown.

The airway epithelium of asthmatics is characterized by thickening, excessive mucus production and inflammatory cell infiltration [3]. In addition, asthmatic airway epithelium exhibits deficient antiviral and repair responses *ex vivo*[4,5]. While transcriptional profiling studies have identified the gene expression profiles characterizing these phenotypic differences [6,7], few studies have investigated the involvement of epigenetic mechanisms in the airway epithelium of asthmatics (e.g. miRNAs [8-10]).

Occurring at cytosines of cytosine-guanine dinucleotides (CpGs), DNA methylation regulates gene expression either directly through inhibition of transcription factor binding or indirectly through recruitment of histone-associated proteins and subsequent chromatin remodeling [1,2]. Studies of DNA methylation in both human and murine models of asthma have focused primarily on circulating cells or the link between environmental exposures, maternal transfer and the development of asthma [1,2]. However, despite representing the initial barrier to environmental exposures linked to asthma development and exacerbation, only a few studies have addressed the role of DNA methylation in the airway epithelium of asthmatics [11-13].

To start to investigate if epigenetic mechanisms could be involved in virus-induced asthma exacerbations, we profiled DNA methylation of primary nasal epithelial cells (NECs) from asthmatic and healthy donors infected *in vitro* with Human Rhinovirus (HRV), the main cause of exacerbations*.* We report DNA methylation patterns associated with asthma as well as differential HRV-induced methylation patterns between asthmatic and healthy subjects. Findings from our study give rise to the hypothesis that epigenetic mechanisms may contribute to the development of virus induced-asthma exacerbations.

## Methods

### Ethics statement

This study was performed at the Allergy, Asthma and Immunology Clinical Research Unit (AAICRU) at Northwestern University. Study design was approved by the Northwestern University Internal Review Board and written informed consent was obtained from all participants prior to study inclusion. This study conformed to the Helsinki Declaration.

### Study recruitment

Individuals with a history of asthma or no chronic respiratory disease (i.e. otherwise healthy) were recruited for the study on a rolling basis via community advertisements. Study exclusion criteria included; smoking in past 6 months, greater than 10 pack-years of lifetime smoking, upper respiratory infection in the previous 4 weeks, stable asthma (i.e. no exacerbation) in previous 4 weeks and any daily controller medications (i.e. steroids). At the AAICRU clinic, demographic data and clinical history were recorded and nasal epithelial cells (NECs) obtained as described previously [14]. Additional clinical characteristics including airway hyperreactivity, lung function and atopic status were determined via methacholine challenge, spirometry and allergen skin test respectively as outlined previously [15].

### Primary nasal cell culture

Nasal epithelial cells were plated in 12-well plates pre-coated with 0.2 mg/mL Type I purified collagen (Vitrogen 100, Advanced Biomatrix Corp.) in complete Bronchial Epithelial Cell Media (BEGM, Lonza) and incubated at 37°C with 5% CO_2_ until 80-90% confluence was achieved. Cells were then trypsinized (0.25% Trypsin EDTA), harvested, split and sub cultured onto collagen coated 24-well plates and maintained in BEGM (passage 1[P1]).

#### Virus infection

When NEC P1 cultures reached 80-90% confluence, cells were infected either with HRV-16 (multiplicity of infection = 2) or phosphate buffered saline (PBS, Mock) in a final volume of 100 μL Bronchial Epithelial Basal Medium (Lonza) for 1 hr at 33°C with mild shaking [14,16]. Infection media was then removed and cells washed twice with warm PBS then 500 μL of fresh BEGM was added and cultures returned to 37°C for 48 hrs.

#### Nucleic acid extraction

Total nucleic acids were isolated from nasal cell cultures using TRI reagent (Life Technologies), phenol/chloroform extraction and ethanol precipitation. Isolated RNA/DNA concentrations were assessed using Nanodrop 1000 (ThermoScientific). DNA from all cultures in Population 1 (Table 1) were used for global methylation profiling. However, only matched mock and infected cultures in Population 1 with sufficient volume (>50 μL) and DNA concentration (>20 ng/μL) were selected for loci-specific profiling in subsequent microarray.

**Table 1:** Demographic and clinical characteristics of study populations

	**Population 1**		**Population 2**		**p value**
	**Healthy (n = 7)**	**Asthma (n = 10)**	**Healthy (n = 6)**	**Asthma (n = 6)**	**Healthy (n = 13) Vs Asthma (n = 16)**
**Female - n (%)**	6 (85.7)	6 (60.0)	1 (16.0)	4 (66.6)	1.00
**Age - median (±SD)**	23 (2.4)	26 (5.9)	20 (0.9)	31 (9.8)	0.02
**BMI - median (±SD)**	22 (3.9)	25 (9.7)	21 (1.5)	23 (5.0)	0.14
**Ethnicity - n (%)**					
White	7 (100.0)	2 (20.0)	3 (50.0)	1 (16.6)	0.01
Hispanic	0	3 (30.0)	0	1 (16.6)	0.10
Black	0	3 (30.0)	0	2 (33.3)	0.04
Asian	0	2 (20.0)	3 (50.0)	1 (16.6)	1.00
Other	0	0	0	1 (16.6)	1.00
**Atopy - n (%)**	1 (14.2)	10 (100)	5 (83.3)	5 (83.3)	0.01
**Allergic Rhinitis - n (%)**	1 (14.2)	9 (90.0)	2 (33.3)	3 (50.0)	0.01
**Chronic Rhinosinusitis - n (%)**	1 (14.2)	1 (10.0)	1 (16.6)	2 (33.3)	1.00
**Albuterol - n (%)**	0	7 (70.0)	0	3 (50.0)	0.004
**FEV1 - median (±SD % predicted)**	101 (9.6)	85 (10.4)	95 (12.3)	92 (22.2)	0.09
**FEV1/FEVC - median (±SD in L)**	0.82 (0.04)	0.78 (0.60)	0.85 (0.03)	0.80 (0.03)	0.05
**PEF - median (±SD in L/min)**	7.06 (1.70)	6.98 (1.50)	7.21 (0.77)	7.47 (1.90)	0.82

Albuterol was the only medication in use within the Asthma group. BMI- body mass index. SD- standard deviation. FEV1 – forced expiratory volume in one second. FVC – forced vital capacity. PEF – Peak expiratory Flow.

### Global and Loci-specific DNA methylation profiling

Global DNA methylation profiling was determined via pyrosequencing of CpGs within genome wide Alu elements as outlined previously [17]. Loci-specific methylation was determined via the Infinium Human Methylation 450 K bead chip microarray (Illumina) and all microarray experiments were performed at the Northwestern University Genomics Core Facility. For microarray analysis, log_2_ ratios of the methylated and unmethylated probes (i.e. M values) were quantile normalized and then subject to linear modeling using Empirical Bayes variance correction [18,19]. Tests of contrast on linear model parameters were used to evaluate pairwise comparisons of interest (Asthma x Healthy pre-HRV, A x H post-HRV, HRV changes in A, HRV changes in H, A x H HRV changes). Statistical analyses of microarray data were performed in R (v2.15.2) using packages from the Bioconductor project [20]. For clarity, genomic loci are reported as gene symbol if the probe target CpG was annotated as gene-associated (e.g. 5′UTR, Body, 3′UTR) or probe identifier if the CpG was associated with another genomic region (e.g. N_Shelf, S_Shelf, Enhancer). Microarray data has been deposited on the Gene Expression Omnibus # GSE52074.

### Principal Component (PCA) and Hierarchical Clustering Analyses

PCAs were performed on covariance matrices of methylation data represented as log_2_ probe M values (n = 389 probes/sample) using PAST (v2.15) [21]. Ranking of component loadings generated for each probe were used to identify the genomic loci influenced by disease-status (principal component 1 - PC1) or virus infection (principal component 2 and 4 - PC2 and PC4). Since no consistent component loading cutoff could be found in the literature, we presented either the top n = 10 most positive or negative loading probes for each respective principal component. Hierarchical clustering of probe M values subsequently identified the patterns of DNA methylation characterizing disease-status or infection. All clustering was performed with Euclidean distance with average linkage using MultiExperiment Viewer v4.4 [22].

### PCR and pyrosequencing

Primers encompassing the two CpGs within SNORA12 (NR_002954.1) were designed using Pyrosequencing™ Assay Design Software (Qiagen). One hundred and fifty nanograms of DNA were bisulfite treated using the EZ DNA Methylation Gold Kit™ (Zymo Research). One microliter of bisulphite converted DNA was subject to PCR employing Hot Star Taq Plus Master Mix (Qiagen) and 10 μM of forward (5′-TGGTGGTTTTTTTTTTGGTATATT-3′) and reverse (5′-Biotin-AATAAAAAAACACCCCTCAACAC-3′) primers. Cycling conditions were; 95°C for 15 min; 95°C for 1 min, 55°C for 1 min, 70°C for 3mins repeated for 40 cycles; 70°C for 10 min then final hold at 16°C. Amplification was confirmed by resolving PCR products on a 1.5% agarose gel. Pyrosequencing employed the Pyromark Gold 96 Kit (Qiagen) and all reactions were run on the PyroMark Q96 MD (Qiagen) using 0.3 μM of sequencing primer (5′-TGGGTTTAATTTTGTTAT-3′). Pyrosequencing data were analyzed and percentage of CpG methylation determined using Pyro Q-CpG™ software (Qiagen).

### Gene expression

One hundred nanograms of RNA were reverse transcribed using SMARTScribe Reverse transcriptase (1U/μL; Clontech), dNTPs and random hexamers (Life Technologies) for 1 hour at 42°C. SNORA12 and GAPDH gene expression were determined using 1 μL of undiluted cDNA and the QuantiFast Multiplex PCR Kit (Qiagen) as per manufacturers protocol. Real time PCR reactions were run on the StepOne™ Real-Time PCR System (Life Technologies).

### Statistics

Significance of changes in Alu methylation and SNORA12 methylation and in gene expression was determined by paired t-test within groups or Mann Whitney *U*-test for comparisons between groups. Correlation analysis for methylation, gene expression and clinical characteristics were determined using Spearman’s Rank Correlation test. Statistical significance between demographic aspects of the Healthy and Asthmatic groups was determined by Fishers Exact test (categorical data) or Mann Whitney *U*-test (numerical data). All statistical analyses were performed using Prism 5 for windows (GraphPad Software Inc).

## Results

### Global DNA methylation

**Figure 1:**
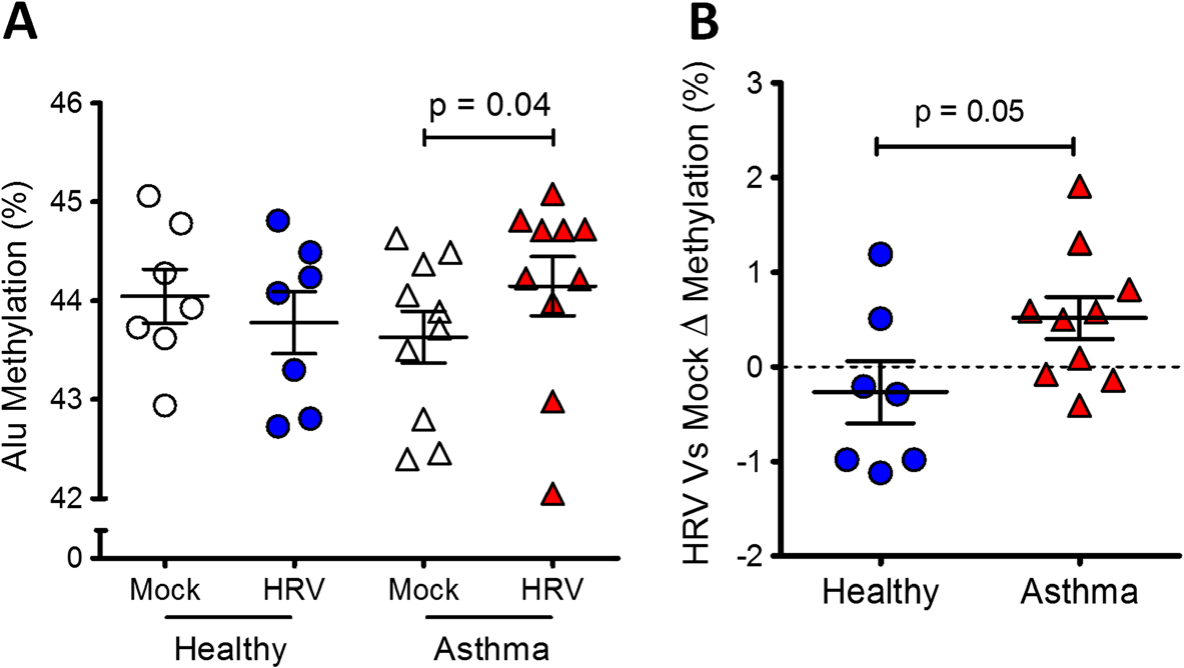
**Global DNA methylation profiling in nasal epithelial cells (NECs) during Human Rhinovirus (HRV) infection. A**. Analysis of CpG methylation within genome wide Alu elements in Mock and HRV infected NECs from Healthy and Asthmatics (Population 1, see Table 1). **B**. Change in Alu methylation in response to virus infection between Healthy and Asthmatics indicating that both disease status and virus infection influenced global DNA methylation in NECs. Mean ± SEM.

To initially investigate the influence of asthma and virus infection on DNA methylation, we determined DNA methylation status of genome wide Alu elements in mock and HRV infected NECs obtained from healthy and asthmatics donors (Population 1, Table 1). Results revealed that HRV infection significantly increased Alu methylation only in cells from asthmatic subjects, indicating that both disease status and virus infection could influence DNA methylation in the airway epithelium (Figure 1).

### Loci-specific DNA methylation

**Figure 2:**
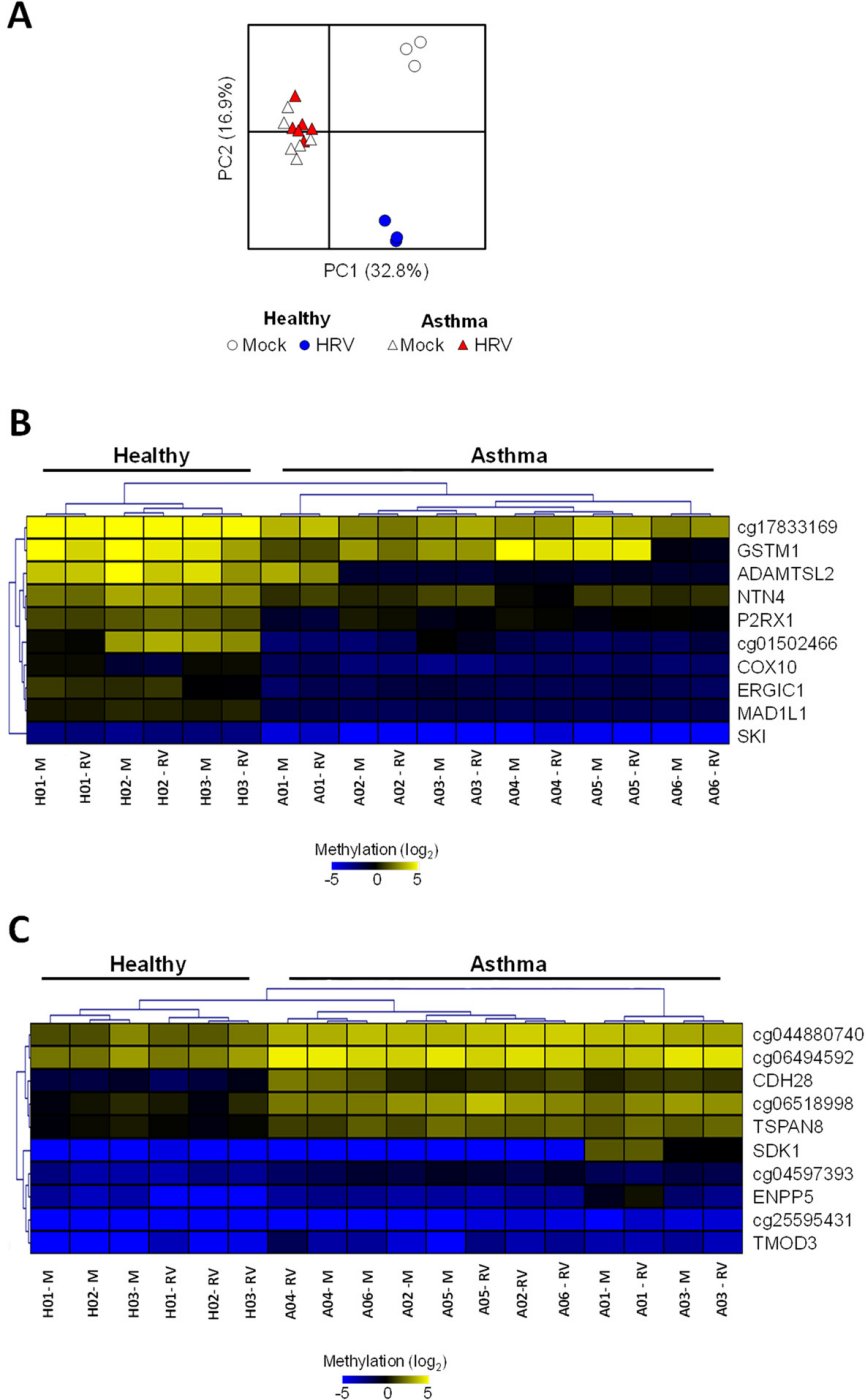
**Influence of disease-status on loci-specific DNA methylation in nasal epithelial cells (NECs). A**. Principal component analysis (PCA) of methylation microarray data set (389 probes/sample) revealed a clear separation between Healthy and Asthmatic NECs along PC1. **B**-**C**. Cluster analysis of genomic methylation loci revealed patterns characterizing Healthy **(B)** and Asthma **(C)** NECs (e.g. Mitotic arrest deficient like-1(yeast)-MAD1L1 for Healthy and Cadherin member 28-CDH28 for Asthma), which were not influenced by virus infection. M-mock, RV- HRV infected.

**Figure 3:**
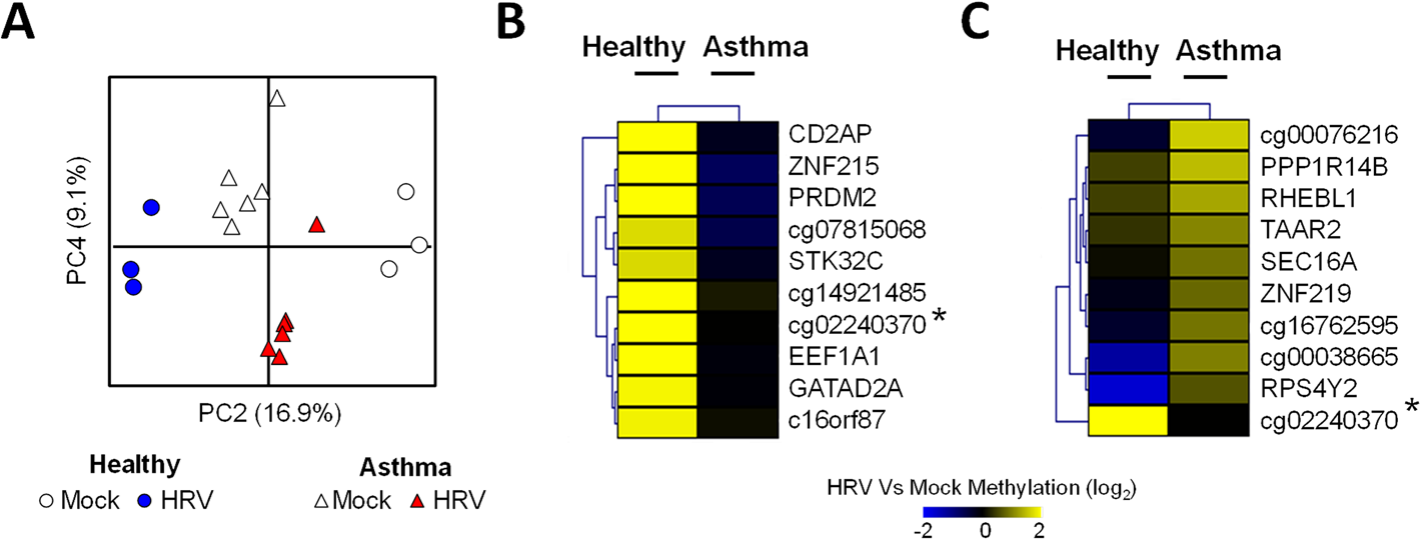
**Influence of Human Rhinovirus (HRV) infection on loci specific DNA methylation in nasal epithelial cells (NECs). A**. Principle components analysis (PCA) of methylation microarray data set (389 probes/sample) revealing a clear separation between infected Healthy and Asthmatic NECs along PC2 and PC4 respectively. **B**-**C**. Genomic loci and methylation pattern characterizing the response to infection in Healthy **(B)** and Asthma **(C)** NECs. Although the majority of loci were group-specific, a common locus (*) was identified between the Healthy and Asthma groups.

To expand and identify the genomic loci influenced the most by disease status and infection, we next used the Infinium Human Methylation 450K microarray to assess loci-specific DNA methylation of matched mock and infected NECs from healthy (H, n = 3) and asthmatic (A, n = 6) subjects (Population 1). Comparative microarray analyses encompassing five possible combinations of conditions/groups (A x H pre-HRV, A x H post-HRV, HRV changes in A, HRV changes in H, A x H HRV changes) revealed differential methylation at several loci including genes and other genomic regions. We then selected the top n = 100 statistically significant loci from each comparison (n = 500 loci in total, p < 0.0001) and in subsequent comparative analysis revealed disease status and infection influenced the methylation of n = 389 distinct loci (Additional file 1: Figure S1).We then used principal component analysis (PCA) to identify DNA methylation patterns (principal components, PCs) that best explain the variation within our methylation data set (n = 389 probes/array sample). PC1 disclosed a methylation pattern that was disease-specific and did not change with HRV infection (PC1; Figure 2A). PC2 and PC4 disclosed methylation changes induced by HRV infection that were specific for healthy and asthmatic subjects, respectively (Figure 3A).

### Disease status influences DNA methylation patterns of NECs

Ranking of positive and negative PC1 loadings identified loci which characterized the Healthy and Asthma NECs, respectively. Hierarchal clustering analysis of microarray data revealed that in addition to being characterized by methylation of distinct loci, the Healthy and Asthma NECs exhibited group-specific patterns of DNA methylation (Figure 2B-C). Remarkably however, the presence of virus infection did not influence either the loci or patterns of methylation characterizing Healthy and Asthma NECs.

### Disease status influences DNA methylation in response to virus infection

**Figure 4:**
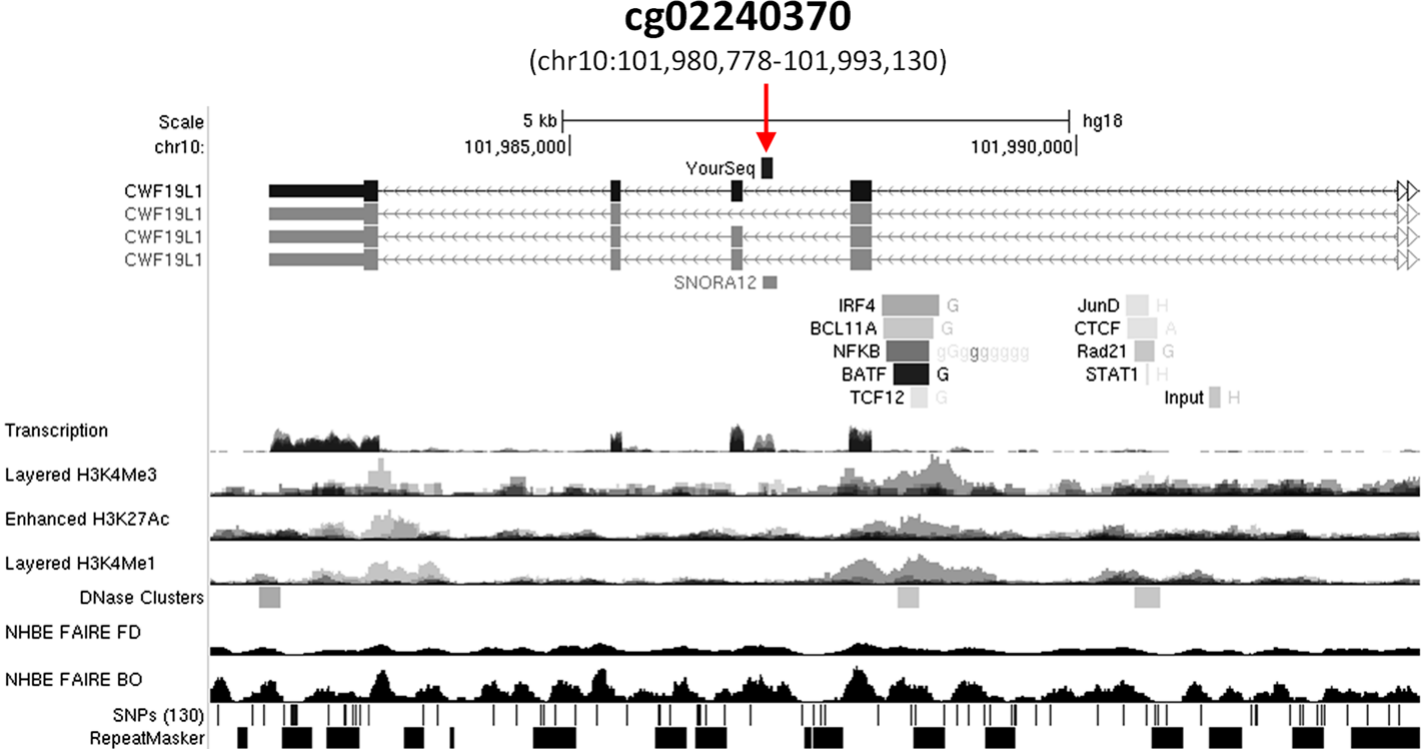
**Genomic features surrounding locus of microarray probe cg02440370.** Screen shot from UCSC genome browser (http://genome.ucsc.edu; assembly NCBI36/hg18) indicating the sequence of probe cg02440370 mapped to chromosome 10 within an intronic region of CWF19-Like 1, Cell Cycle Control (CWF19L1) and directly in the location of SNORA12 (red arrow). A number of transcription factors associated with antiviral gene expression (e.g. IRF4) had bindings sites located upstream of SNORA12. In addition, chromatin structures permissible to transcription (e.g.H3K4me3) were also located within the same region of transcription factor binding. Data tracks are derived from the ENCODE project.

Ranking of negative loadings of PC2 and PC4 identified unique loci whose methylation changed after HRV infection in NECs from healthy and asthmatic subjects respectively (Figure 3B-C). Interestingly, methylation of a locus represented by probe cg02240370 was common in both groups. Further investigation revealed that the CpG represented by probe cg02240370 mapped to Chromosome 10 in the location of the small nucleolar RNA, H/ACA box 12 (SNORA12, Figure 4). Binding sites of a number of transcription factors associated with antiviral gene expression (e.g. interferon regulatory factor 4-IRF4, nuclear factor kappa beta-NFκB) and chromatin modifications permissible to transcription (e.g. histone 3 lysine 4 trimethylation-H3K4me3) were also located upstream of SNORA12. Considering these findings were highly relevant to virus infection, we subsequently focused on investigating the effect of DNA methylation on SNORA12 gene expression.

### DNA methylation affects SNORA12 gene expression in response to virus infection

**Figure 5:**
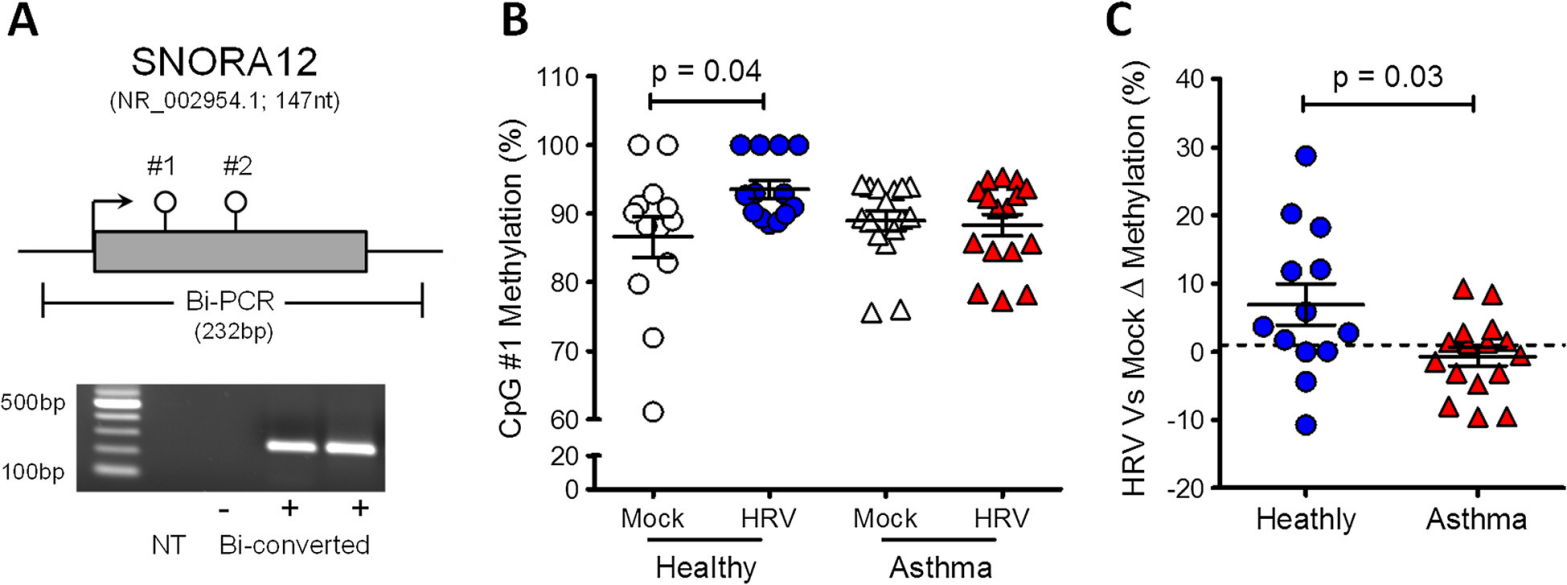
**DNA methylation status of SNORA12 in Mock or Human Rhinovirus (HRV) infected nasal epithelial cells (NECs). A**. Schematic of SNORA12 pyrosequencing assay design. In addition to the CpG targeted by probe cg02440370 (#1), a second CpG (#2) was identified within the coding region. Both CpGs were amplified in bisulphite-specific PCR (Bi-PCR). **B**. Analysis of SNORA12 methylation in both study populations (see Table 1) revealed a significant increase at CpG#1 within the Healthy NECs during infection. No changes were observed at CpG#2 (data not shown). **C**. Change in CpG#1 methylation in response to infection for Healthy and Asthmatic NECs. Mean ± SEM ,*p = <0.05. NT – no template control.

**Figure 6:**
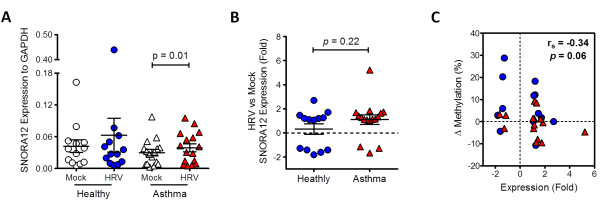
**Relationship between SNORA12 methylation and gene expression in Mock or Human Rhinovirus (HRV) infected nasal epithelial cells (NECs). A**-**B**. Analysis of SNORA12 gene expression in both study populations (see Table 1) revealed a significant increase in infected NECs within the Asthmatic group **(A)**. However no difference in SNORA12 gene expression existed between Healthy and Asthmatics in response to infection **(B)**. **C**. Correlation analysis between SNORA12 methylation and gene expression within both study groups in response to infection. Mean ± SEM, r_s_-spearmans rho.

Using a pyrosequencing and real-time PCR approaches, we determined the methylation status and gene expression of SNORA12 in our initial cultures and an additional population of mock and infected NECs from Healthy (n = 6) and Asthmatic (n = 6) donors (Population 2, Table 1). Similar to microarray results, we identified an increase in SNORA12 methylation in response to infection exclusively within the Healthy group (Figure 5). Conversely, SNORA12 gene expression was significantly increased during infection within the asthmatics (Figure 6A-B). Correlation analysis then revealed a collective negative trend was present between SNORA12 expression and DNA methylation in response to virus infection (Figure 6C).

### Changes SNORA12 gene expression during infection are associated with lung function

Finally, we determined if changes in SNORA12 gene expression or methylation were associated with any of the clinical characteristics of our study populations (Table 2). We observed opposing relationships between SNORA12 gene expression and measurements of lung function within the Healthy and Asthmatics groups (FEV1/FVC and FEV1% predicted respectively). However no relationship existed between changes in SNORA12 DNA methylation and the clinical characteristics of our study populations.

**Table 2:** Correlation between changes in SNORA12 gene expression and DNA methylation during infection with clinical characteristics of our study populations

	**Healthy (n = 13)**				**Asthma (n = 16)**				**Both Groups**			
	**Expression**		**Methylation**		**Expression**		**Methylation**		**Expression**		**Methylation**	
	**r**_**s**_	**p value**	**r**_**s**_	**p value**	**r**_**s**_	**p value**	**r**_**s**_	**p value**	**r**_**s**_	**p value**	**r**_**s**_	**p value**
Atopy	0.16	0.59	0.12	0.69	0.20	0.47	0.17	0.53	0.26	0.18	-0.09	0.63
FEV1 (% predicted)	-0.36	0.22	0.37	0.21	0.71	0.002	0.00	0.99	0.11	0.59	0.27	0.15
FEV1/FVC (L)	**-**0.57	0.04	-0.09	0.76	0.43	0.10	0.23	0.39	-0.12	0.55	0.29	0.13
PEF (L/min)	-0.08	0.80	-0.08	0.79	-0.13	0.64	-0.04	0.88	-0.08	0.68	0.00	1.00
Nasal Allergies	-0.22	0.50	0.39	0.21	0.08	0.78	-0.02	0.95	0.13	0.51	-0.17	0.39
Chronic Rhinosinusitis	0.06	0.84	0.32	0.30	-0.15	0.58	-0.06	0.84	-0.05	0.81	0.10	0.61
Albuterol use	n/a	n/a	n/a	n/a	-0.29	0.28	0.07	0.80	-0.01	0.96	-0.21	0.27

FEV1 – forced expiratory volume in one second. FVC – forced vital capacity. PEF – Peak expiratory Flow. r_s_ – spearmans rho.

## Discussion

Despite exhibiting distinct transcriptional and phenotypic characteristics, only a limited number of studies have focused on epigenetics such as and microRNAs [8-10] and DNA methylation in the airway epithelium of asthmatics [11-13]. Baccarelli *et al.* investigated global (via Alu) and gene-specific DNA methylation in NECs from asthmatic children and found an association between inflammatory gene promoter methylation and exhaled nitric oxide [13]. Using microarray, Stefanowicz *et al.* investigated bronchial brushings from healthy, atopic and non-atopic asthmatic children and identified atopic-status primarily influenced DNA methylation [11]. Likewise, Kim *et al.* found more differentially methylated regions between atopic and non-atopic asthmatics than between healthy and asthmatics adults [12]. Our own study has determined that NECs from asthmatic adults were characterized by a distinct profile and pattern of DNA methylation (Figure 2). However, given our asthmatics were almost exclusively atopic (15/16 – 93%; Table 1), results from our study may have also reflected the influence of allergic airway disease on DNA methylation.

We found that underlying airway disease was associated with a specific DNA methylation pattern that was not altered by HRV infection. Although only a few studies have been conducted, it seems that atopy is associated with specific DNA methylation in both children and adults with asthma, suggesting either an inherited pattern, or a consequence of allergic inflammation. Indeed, Interlukin-13 has been shown to alter DNA methylation in the airway epithelium [8,23] and even remodeling cytokines (e.g. Transforming growth factor-β, Fibronectin) can modulate histone methylation (e.g. H3K4me3) [5,24,25]. However, both allergic airway disease and remodeling involve complex interactions between many cell types and effects of select cytokines on DNA methylation maybe cell type-specific [26]. Since we studied upper airway epithelial cells, our study design could not distinguish effects of allergic rhinitis from those of asthma. Moreover, our findings may have also been confounded by the diversity in age and ethnicity within our study populations (Table 1). Future work dissecting the effects of asthma, Th2 and remodeling cytokines on DNA methylation in both the upper and lower airway epithelium will therefore be important to this field.

Our finding that HRV infection alters epithelial DNA methylation adds to the growing and remarkable evidence that environmental factors change human DNA. It is now known that environmental exposures that increase risk of developing asthma such as smoking and air pollution can also alter DNA methylation [27,28]. We showed that respiratory virus infection can influence both global and loci-specific DNA methylation in NECs. Analysis of Alu methylation indicated increased global methylation occurred in NECs from the asthmatic group in response to virus infection (Figure 1). Interestingly, production of interferon can be dependent on transcription factor binding within Alu elements [29] and some asthmatics are characterized by deficient interferon production in response to virus infection [4]. Determining the methylation status of Alu elements proximal to interferon genes may reveal an epigenetic basis for the decreased interferon production in asthmatics during virus infection.

While previous studies have indicated that respiratory viruses can affect the methylation and expression of select cytokines [30,31], our study found that DNA methylation profiles in response to infection of NECs from both healthy and asthmatic subjects were not dominated by loci associated with proinflammatory or antiviral immune responses (Figure 3). Indeed, the differential methylation of both genes and intergenic CpGs impeded the identification of any dominant biological process by gene enrichment analysis (data not shown). These findings suggest that DNA methylation may not represent an important regulatory mechanism of immune-associated loci/genes in airway epithelial cells. However, it is conceivable that changes in DNA methylation of immune associated loci occurred earlier than our study time point (i.e. <48 hrs) or immune related process might have been identified if less stringent criteria of loci selection from PCA was conducted (e.g. top n = 100 most negative/positive loading loci per component). Future studies investigating temporal infection or effects of toll-like receptor agonists and inflammatory cytokines (i.e. interferon, CXCL family members) would address these issues and reveal the dynamics of DNA methylation in response to infection.

Although the response to virus was characterized by methylation of distinct loci in both the Healthy and Asthmatic NECs, principal component analysis pointed to a common locus in both groups, where SNORA12 is located (Figure 3). SNORA12 is a member of the small nucleolar RNA, H/ACA box family which encompass 103 non coding RNAs involved in diverse cellular processes including modification of ribosomal RNA, messenger RNA splicing, and maintenance of genome integrity [32]. While the function of SNORA12 in HRV infection remains to be determined, we observed changes in SNORA12 gene expression during virus infection (Figure 5). Interestingly, binding sites of a number of transcription factors associated with antiviral gene expression (e.g. NFκB, IRF4) and chromatin architecture permissive to transcription were located up stream of SNORA12 (e.g. H3K3me3, Figure 4). Given virus infection and other inflammatory diseases have been shown to affect the expression of snoRNA’s and other non coding RNAs [33,34], these findings suggest that SNORA12 is a component of the antiviral or inflammatory processes within the airway epithelium.

Despite being common in response to infection, we observed that SNORA12 methylation differed in atopic asthmatic and healthy subjects (Figures 5 and 6). Although no significant relationship between methylation and gene expression was evident, the trend observed supports our previous findings that underlying respiratory disease can influence gene expression in response to virus infection [35] and reveals for the first time that this influence is likely mediated via epigenetic mechanisms. Furthermore, because we observed a relationship between SNORA12 gene expression and lung function within our study groups, these findings would suggest that methylation-mediated changes in gene expression caused by underlying respiratory disease may also have functional consequences. Comprehensive investigation of epigenetic mechanisms prior to, during and after virus induced exacerbation could therefore reveal if component(s) of the epigenome contribute to the development of exacerbation.

## Conclusion

In the present study we have determined that human rhinovirus infection causes different changes in global and loci-specific DNA methylation in nasal epithelial cells from asthmatic subjects compared to cells from healthy subjects. We also found group-specific DNA methylation patterns that were not affected by HRV infection. While the focus of this study exclusively on the upper airways may limit our current findings, these distinct responses to HRV infection within the methylome may in the future shed light on epigenetic mechanisms that play a role in virus-induced asthma exacerbations.

## Abbreviations

NEC: Nasal epithelial cells; HRV: Human rhinovirus; SNORA12: Small nucleolar RNA, H/ACA box 12; PC: Principal component.

## Competing interests

All authors declare no competing interests.

## Authors’ contributions

PCA, MBS, SF and PM designed and conceived the study. PCA and JQ recruited and characterized study volunteers. SF, JS and AB conducted cell culture. FFC, EFV, MB and HX conducted global methylation experiments and analysis. DMS and PM conducted microarray analysis. PM, FFC and JJC conducted bisulphite-PCR. PM and PCA wrote the manuscript. All authors read and approved the final manuscript.

## Pre-publication history

The pre-publication history for this paper can be accessed here:

http://www.biomedcentral.com/1755-8794/7/37/prepub

## Supplementary Material

Additional file 1: Figure S1 Summary or microarray analysis and identification of distinct loci influenced by disease staus or virus infection. HRV – Human Rhinovirus. Click here for file
