# Supplementary figures and images for: Human rhinovirus infection causes different DNA methylation changes in nasal epithelial cells from healthy and asthmatic subjects
Source: BMC Med Genomics. 2014 Jun 19;7:37. doi: 10.1186/1755-8794-7-37 (PMC4080608; doi:10.1186/1755-8794-7-37)

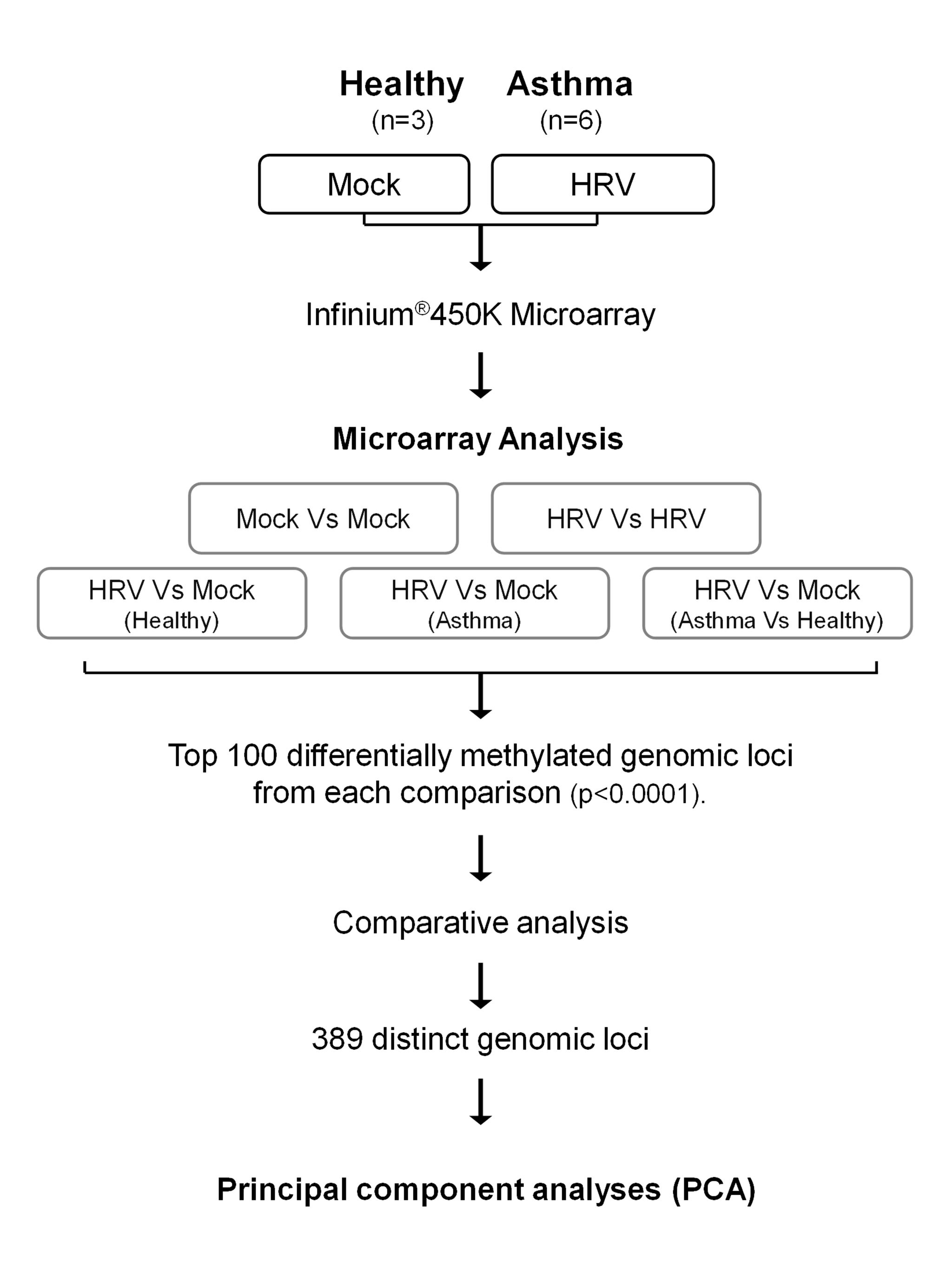

Supplement: Additional file 1: Figure S1 — Summary or microarray analysis and identification of distinct loci influenced by disease staus or virus infection. HRV – Human Rhinovirus. [file 1755-8794-7-37-S1.tiff]
